# A systematic review of depression and anxiety in medical students in China

**DOI:** 10.1186/s12909-019-1744-2

**Published:** 2019-09-02

**Authors:** Ying Mao, Ning Zhang, Jinlin Liu, Bin Zhu, Rongxin He, Xue Wang

**Affiliations:** 1 School of Public Policy and Administration, Xi’an Jiaotong University, Xi’an, 710049 China; 2 Department of Public Policy, City University of Hong Kong, Hong Kong, 999077 China

**Keywords:** Depression, Anxiety, Medical students, Systematic review, Meta-analysis

## Abstract

**Background:**

Medical students in China face severe depression and anxiety because of their difficult circumstances, such as the long length of schooling, academic pressure, and the stress of clinical practice. Although there have been many empirical studies about depression or anxiety in medical students in China, no previous studies have conducted a related systematic review about this topic in English. This analysis can convey the general findings from China to other areas of the world.

**Methods:**

A systematic review and meta-analysis of depression or anxiety in medical students and related determinants were conducted. Three Chinese and three English databases were searched for the review, with no restrictions on language. Articles published between January 1, 2000 and April 1, 2018 were included.

**Results:**

Twenty-one articles investigating a total of 35,160 individual Chinese medical students were included in this review. The prevalence of depression ranged from 13.10 to 76.21% with a mean of 32.74%, and the prevalence of anxiety ranged from 8.54 to 88.30% with a mean of 27.22%. Based on the meta-analysis, gender, grade level, residence, satisfaction with current major and monthly household income per capita were significantly associated with depression. Grade level and satisfaction with current major were significantly associated with anxiety. Other risk factors were identified and described using a narrative approach.

**Conclusion:**

The mean prevalence of depression was 32.74% amongst medical students in China, whereas the mean prevalence of anxiety was 27.22%. The determinants of depression and anxiety included individual factors, social and economic factors, and environmental factors. More measures should be taken towards at-risk medical students based on the identified risk factors.

**Electronic supplementary material:**

The online version of this article (10.1186/s12909-019-1744-2) contains supplementary material, which is available to authorized users.

## Background

Depression and anxiety are amongst the most frequent mental disorders. Mental disorders have received increasing global attention because of their negative effects on working ability and the performance of people. As the core of the health system in all countries [1], medical workers are suffering from depression or anxiety resulting from their deteriorative treatment environment (i.e., workplace violence) [2], overwork [3], burnout [4], huge academic pressure [5], declining job satisfaction [6], etc.

Similarly, as the successors of medical workers, medical students have reported experiencing a higher level of depression or anxiety compared to their peers [7]. Medical students should be fully aware of the difficulties before entering into the job market. During the study portion of medical school, which is seemingly relaxed and enjoyable, medical students still experience huge pressure, such as the stress of the long length of schooling, academic pressure, the stress of clinical practice, etc. [8]. 27.2% of medical students, as reported by Rotenstein et al., were subjected to depression or depressive symptoms in 47 countries. [9]. Different prevalence results have been found in many other countries and regions [10–12]. However, the determinants of depression and anxiety are similar, according to previous studies [13, 14].

Several studies have explored the effects of various risk factors of depression and anxiety in medical students, such as age [9–11], gender [5, 15], grade [4], ethnicity [14, 16], residence [17, 18], current major [19, 20], school [21], learning stage [22, 23], length of schooling [24], being an only child [18–21], rating of school [22, 23, 25], satisfaction with current major [26], attitude towards future career [27, 28], academic pressure [29], smoking addiction [26], alcoholism [26], school loans [21, 25], clinical internship [30], the ability to deal with interpersonal relationships [19, 22], parents’ education [24], parental occupational status [31, 32], family financial status [27–29], social support factors and other factors [33].

In the Chinese context, medical students also suffer from severe mental diseases, particularly depression and anxiety, which mainly caused by the huge pressure during school [34–36], the extremely long length of schooling [7], and worries about horrible working conditions, such as the stressful relationship between patients and doctors [37]. Many empirical studies have been conducted in China; however, most of them were published in Chinese journals, and no related systematic reviews have been found.

Based on the above findings, this study aimed to examine the prevalence of depression or anxiety amongst medical students in China by conducting a systematic review and a systematic research study about their determinants.

## Methods

A systematic review was conducted based on studies published in English and Chinese databases from January 1, 2000 to April 1, 2018, following the Preferred Reporting Items for Systematic Reviews and Meta-Analyses (PRISMA) guidelines [38]. We searched the following English databases: PubMed, EMBASE and Cochrane Library; we also searched the following Chinese databases: China National Knowledge Internet Database (CNKI), Wanfang database, and China Biology Medicine disc (CBM).

### Search strategy and selection criteria

For the three English databases, i.e., PubMed, EMBASE and Cochrane Library, we used a search strategy based on a combination of the following terms: [(undergraduate medical student) OR (medical student) OR (Trainee doctor) OR (student doctor)] AND [(Depression) OR (Major depressive disorder) OR (Depressive disorder) OR (Anxiety)] AND [(China) OR (Chinese)].

For the three Chinese databases, i.e., China National Knowledge Internet Database (CNKI), Wanfang database, and China Biology Medicine disc (CBM), we used a combination of the following terms: [zhong-guo OR zhong-guo-de] (China/Chinese) AND [yi-xue-sheng OR shi-xi-yi-sheng OR yi-xue-zhuan-ye-xue-sheng] (Medical students/Trainee doctor/undergraduate medical students) AND [yi-yu OR jiao-lv] (Depression/anxiety).

The search strategy is shown in Additional file 1.

The eligibility criteria included the following: (1) Time and location: the systematic review included published articles (without language restrictions) conducted in China between January 1, 2000 and April 1, 2018. (2) Study participants: the study population was comprised of medical students. (3) Studies that measured the degree of depression or anxiety using validated assessment tools. (4) Studies that analysed related determinants of depression or anxiety in medical students. (5) Studies that reported on the pressure of medical students under emergency or special circumstances (earthquakes, severe acute respiratory syndromes, etc.) were excluded. (6) Only cross-sectional studies were included.

### Data analysis

**Fig. 1:**
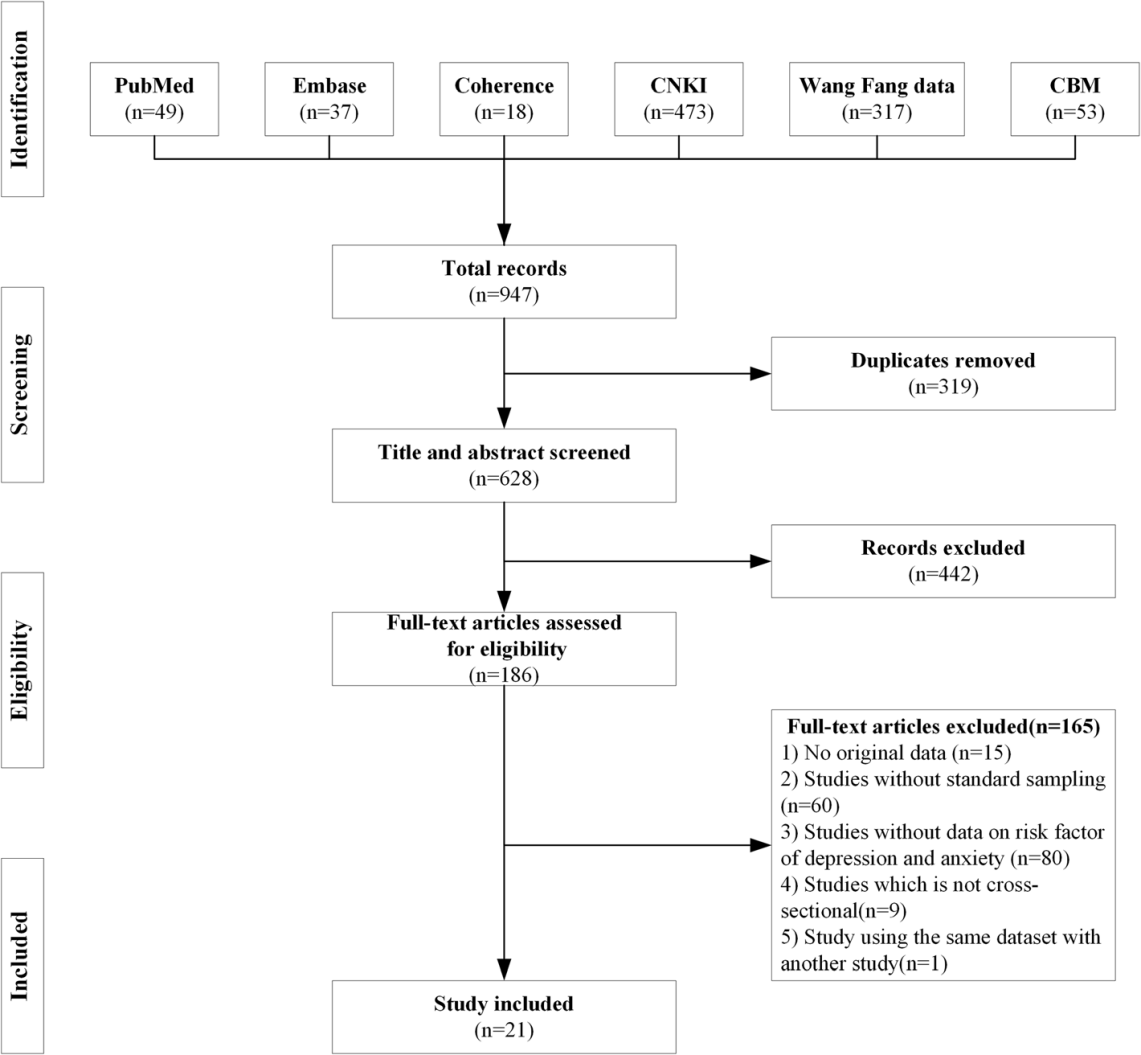
Flow diagram of study selection

Two independent reviewers participated in the data extraction by screening the acquired studies at the same time, according to the flow diagram (shown in Fig. 1). Disagreements were resolved through discussions between the two reviewers and full group consensus. The basic information extracted from each included study contained the first author, year of publication, locations, participants, mental problem types, sample size, qualified rate, assessment tools, disease incidence cases, incidence rates and the number of references (shown in Table 1).

**Table 1:** Tools used to assess medical students’ mental health and determinants

Assessment tools	Assessment tool (abbreviated)	Standard	Studies (n)	Ref. No
Depression				
Beck Depression Inventory	BDI	A score of ≥14 out of 84 indicates depression	3	[39]
Centre for Epidemiologic Studies Depression Scale	CES-D	A score of ≥15 out of 60 indicates depression	4	[40]
Zung self-rating Depression Scale	SDS	A score of ≥40 out of 80 indicates depression	8	[41]
Anxiety				
Symptom Checklist 90	SCL-90	A score of ≥1.8 out of 5 indicates anxiety	2	[42]
Beck Anxiety Inventory	BAI	A score of ≥5 out of 63 indicates anxiety	1	[43]
Zung Self-rating Anxiety Scale	SAS	A score of ≥40 out of 80 indicates anxiety	6	[44]
Hamilton Anxiety Rating Scale	HAMA	A score of ≥14 out of 56 indicates anxiety	3	[45]
Determinants				
Perceived Social Support Scale	PSSS	–	2	[46]
Eysenck Personality Questionnaire	EPQ	–	2	[47]
Life Events Scale	LES	–	1	[48]
China College Student Adjustment Scale	CCSAS	–	1	[49]
Life Orientation Test-Revised	LOT-R	–	1	[50]
Resilience Scale-14	RS-14	–	1	[51]
Adult Dispositional Hope Scale	ADHS	–	1	[52]
Social Support Rating Scale	SSRS	–	2	[53]
Family APGAR Index	APGAR	–	1	[54]
Student-life Stress Inventory	SLS	–	1	[55]
Coping Style Questionnaire	CSQ	–	1	[56]
The Medical Outcomes Study 36-Item Short-form Health Survey	SF-36	–	1	[57]
Beck Hopeless Scale	BHS	–	1	[58]
Adolescent Self-Rating Life Events Check List	ASLEC	–	1	[59]
The Pittsburgh Sleep Quality Index	PSQI	–	1	[60]

The quality of evidence was assessed using the Grading of Recommendations: Assessment, Development, and Evaluation (GRADE) approach [61] for observational studies. The quality assessment standard of the studies evaluated the representativeness of the sample, sample size, non-respondents, ascertainment of the exposure, comparability of subjects in different outcome groups, assessment of the outcome, and use of appropriate statistical test (shown in Table 2). The maximum score was 7, a score of 5–7 indicated good quality, a score of 3–4 indicated medium quality, and a score of 1–2 indicated poor quality. Studies with medium and good quality were included in our analysis.

**Table 2:** Quality scores assessing the risk of bias using a modified Newcastle-Ottawa scale

Study type: Cross-sectional; Score: 1 = achieved, 0 = not achieved								
Authors	Representativeness of the sample	Sample size	Non-respondents	Ascertainment of the exposure	Comparability of subjects in different outcome groups (control for confounding)	Assessment of the outcome	A statistical test is appropriate	Total score
Lin, 2018 [62]	0	1	1	1	1	1	1	6
Li, 2017 [63]	1	1	1	1	1	1	1	7
Pan, 2016 [64]	1	1	0	1	1	1	1	6
Li, 2015 [65]	0	1	1	1	1	1	1	6
Li, 2015 [66]	1	1	1	1	1	1	1	7
Zhai, 2006 [67]	0	1	1	1	1	1	1	6
Guo, 2014 [35]	0	1	1	1	1	1	1	6
Ren, 2013 [68]	0	1	1	1	1	1	1	6
Ruan, 2011 [69]	0	1	0	1	1	1	1	5
Sun, 2011 [70]	1	1	1	1	1	1	1	7
Feng, 2010 [71]	0	1	1	1	1	1	1	6
Shang, 2009 [72]	1	1	1	1	1	1	1	7
Li, 2009 [73]	0	1	1	1	1	1	1	6
He, 2009 [74]	0	1	1	1	1	1	1	6
Liu, 2009 [75]	0	1	1	1	1	1	1	6
Liang, 2007 [76]	0	1	1	1	1	1	1	6
Qu, 2014 [34]	0	1	1	1	1	1	1	6
Yang, 2005 [77]	0	1	1	1	1	1	1	6
Zhou, 2003 [78]	0	1	1	1	1	1	1	6
Yu, 2001 [79]	0	1	0	1	1	1	1	5
Wu, 2000 [80]	1	1	1	1	1	1	1	7

### Synthesis of results

This study adopted the framework of the social determinants of health (SDH), which were proposed by the 66th World Health Assembly of the World Health Organization (WHO), to synthesize the results [81]. “The SDH are the conditions in which people are born, grow, work, live, and age, and the wider set of forces and systems shaping the conditions of daily life. These forces and systems include economic policies and systems, development agendas, social norms, social policies and political systems” [82]. According to the framework, apart from the direct influencing factors, there are other factors in a social structure such as class, power and wealth. These factors were collectively referred to as environmental characteristics. Mental health is also a part of overall health. Previous scholars have identified an association between mental health and social determinants [83, 84]. Based on the conceptual framework and the WHO guidelines of the determinants of mental health, the determinants of medical students’ mental health (depression or anxiety) were categorized into three parts: individual factors (IFs) (including biological factors, psychological factors, and behavioural factors), social and economic factors (SEFs) (including relationship with others and economic status) and environmental risk factors (EFs) (including inequality, racism, discrimination, refugee, war and immigrants).

### Statistical analysis

Two main outcomes were evaluated. The first outcome was the prevalence and determinants of depression analysis, whereas the second outcome assessed the prevalence and determinants of anxiety. Not all the determinants were included in the meta-analysis. It was required that were the same or could be merged into the same category. The variable screening process is shown in Additional file 1. In this study, the different variables were transformed into binary variables; meanwhile, the included determinants were those reported in at least two articles.

The determinants included in the meta-analysis were those reported in at least three articles. In the meta-analysis, the significance of the pooled odds ratio (OR) was determined by the Z-test, with a *P* < 0.05 considered statistically significant. The Q statistic was calculated to estimate the heterogeneity, and *P* ≤ 0.10 was considered statistically significant [85]. A fixed-effect model was used to compute the summary risk estimate. We assessed the possibility of publication bias for the studies included in the meta-analyses with Egger’s linear regression test, which was used to quantitatively evaluate the asymmetry. In this study, all the significance levels were set at *p*-value < 0.05. The null hypothesis is that there is no difference between variable values. The null hypothesis is rejected when *p* < 0.05 and not rejected when *p* > 0.05.

Forest maps were created to illustrate the results and significance of the included articles. In the plane Cartesian coordinate system, the forest map uses a vertical equivalent line as the centre and describes the results and confidence intervals of each included study via multiple lines parallel to the horizontal axis. The forest map uses a diamond, which is the black quadrilateral shown in the map, to describe the results and confidence intervals of multiple studies.

**Fig. 2:**
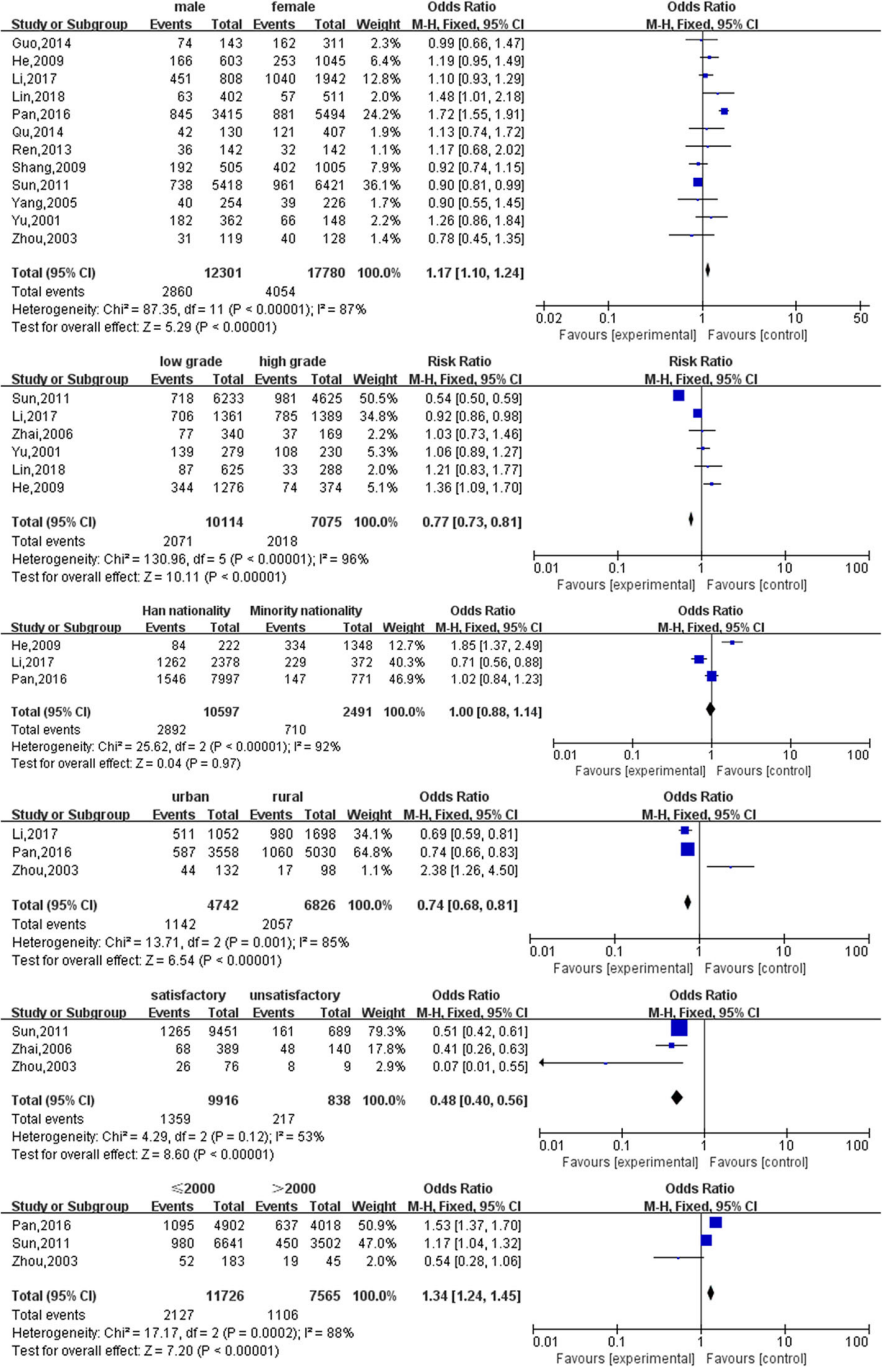
Forest plots of depression-related factors

Using gender (shown in Fig. 2) as an example, there was no significant difference between males and females, as shown by the horizontal lines crossing the vertical line. When horizontal lines were located to the left of the vertical line, females displayed more depressive symptoms than males. When the horizontal lines were located to the right of the vertical line, the reverse case was true.

If it was impossible to perform a quantitative synthesis and conduct a meta-analysis, we used the narrative approach and descriptive statistics, which reported the same determinants, and compared their associations with depression or anxiety.

All statistical analyses were performed using Stata 13.0 (Stata Corp, College Station, TX, USA) and RevMan 5.3 (The Cochrane Collaboration, Oxford, UK).

## Results

### Search results

The study selection process was shown in Fig. 1. This study was conducted according to the PRISMA flow diagram and included four stages: identification, screening, eligibility and inclusion. The initial database search identified 947 articles; after removing duplicate records, 628 remained. The two reviewers examined the remaining articles by screening the titles and abstracts. In total, 186 articles were included in the full-text review. Amongst these studies, 15 articles were eliminated because they lacked original data, 60 articles were removed because they lacked standard sampling, 80 articles were removed because they did not analyse the determinants of depression or anxiety, nine articles were not cross-sectional, and two articles used the same database. Finally, 21 studies were included in this study.

### Analysis of the included articles

Table 1 presents the full name and abbreviated name of the assessment tools utilized in the 21 articles. The included articles utilized three depression assessment tools, four anxiety assessment tools, and 15 determinants assessment tools. Table 3 presents the basic features of the individual studies. The 21 articles contained 35,160 individual participants distributed in the 23 provinces. Two articles included an analysis of different cities, and the others investigated one individual city.

**Table 3:** Characteristics of the 21 included studies

Authors	Location	Participants	Mental problem	Number (Qualified rate %)	Assessment tools	Incidence rate, N (%)	Ref. No.
Lin, 2018	Wuhan	Medical undergraduate students	Depression	913 (86.95)	SDI & PSSS & EPQ & LES & CCSAS	120 persons (13.10)	[62]
Li, 2017	seven provinces	Undergraduate students in universities of traditional Chinese medicine	Depression	2750 (89.29)	CES-D	993 persons (36.11)	[63]
Pan, 2016	23 provinces	Medical undergraduate students	Depression	9010 (77.57)	BDI	1793 (19.9)	[64]
Li, 2015	Henan	Medical undergraduate students	Anxiety	848 (94.20)	SAS	172 (20.30)	[65]
Li, 2015	Henan	Medical undergraduate students	Anxiety	1494 (95.34)	SLS & SAS	390 (26.10)	[66]
Zhai, 2006	Anhui	Medical undergraduate students	Depression & Anxiety	576 (95.68)	SDS & HAMA	175 (30.38) and 232 (40.28)	[67]
Guo, 2014	Xining	Medical undergraduate students	Depression	473 (92.75)	SDS	246 (52.00)	[35]
Ren, 2013	Harbin	Medical undergraduate students	Depression	279 (97.21)	SDS	62 (21.60)	[68]
Ruan, 2011	Lanzhou	Medical undergraduate students	Anxiety	530 (not stated)	SAS	133 (25.09)	[69]
Sun, 2011	Anhui	Medical undergraduate students	Depression & Anxiety	10,374 (92.05)	SAS	1743 (16.80) and 1463 (14.10)	[70]
Feng, 2010	Ningxia	Medical undergraduate students	Anxiety	967 (98.17)	SAS	276 (28.54)	[71]
Shang, 2009	Yinchuan	Medical undergraduate students	Depression	1510 (94.38)	SDS	593 (39.27)	[72]
Li, 2009	Guangzhou	Medical undergraduate students	Depression	206 (82.40)	CES-D	157 (76.2)	[73]
He, 2009	Yunnan	Medical undergraduate students who were minorities	Depression & Anxiety	1655 (97.30)	SCL-90	1.79 ± 0.53 and 1.65 ± 0.44	[74]
Liu, 2009	Guangzhou	Medical undergraduate students	Anxiety	195 (97.50)	HAMA	133 (83.6)	[75]
Liang, 2007	Guangxi	Medical college students in freshman year	Anxiety	615 (90.84)	HAMA	113 (16.6)	[76]
Qu, 2014	Shenyang	Medical undergraduate students	Depression & Anxiety	509 (97.88)	SDS & SAS & EPQ & PSSS & ASLEC	110 (22.40) and 44 (9.03)	[34]
Yang, 2005	Weifang	Medical undergraduate students	Depression & Anxiety	500 (97.09)	SDS & SAS & PSQI	83 (16.60) and 43 (8.60)	[77]
Zhou, 2003	Guangzhou	Medical undergraduate students	Depression	176 (95.65)	SDS	71 (40.43)	[78]
Yu, 2001	Fujian	Medical undergraduate students	Depression	509 (not stated)	CES-D	156 (30.5)	[79]
Wu, 2000	Datong	Medical college students	Depression	1071 (95.20)	SDS	461 (43.00)	[80]

Seven provinces menn Beijing, Liaoning, Gansu, Guangxi, Yunnan, Henan, Hubei. 23 provinces mean Heilongjiang, Liaoning, Jilin, Beijing, Hebei, Henan, Shandong, Shanxi, Shaanxi, Nei Monggol, Shanghai, Jiangsu, Anhui, Zhejiang, Fujian, Guangdong, Guangxi, Yunnan, Jiangxi, Gansu, Ningxia, Sichuan, Chongqing

All 21 articles were conducted in medical colleges or universities (two in medical colleges and 19 in medical universities). One study focused on undergraduate students in a Chinese traditional medical school, one article analysed undergraduate students who were minorities, one article evaluated the mental health of freshmen students, and two articles studied college medical students. The other studies focused on undergraduate medical students in general.

Ten articles analysed the prevalence and determinants of depression [35, 62–64, 72, 73, 78–80], six articles analysed the prevalence and determinants of anxiety [65, 66, 69, 71, 75, 76], and five articles included an analysis of both depression and anxiety [34, 67, 70, 74, 77].

Fifty-seven determinants were extracted, which were determined based on their associations with depression or anxiety in medical students in the 21 included studies (shown in Additional file 1). According to the SDH and WHO guidelines, the determinants of mental health [83] were categorized into three groups: (1) 30 individual factors: gender, grade, age, ethnicity, residence, current major, type of school, school location, learning stage, length of schooling, number of siblings, rating of school, satisfaction with current major, attitude towards future career, academic pressure, clinical internship, retaking college entrance examination as a senior, personal character score, adaptability factor score, interest in current major, satisfaction with school life, satisfaction with accommodations, sleeping conditions, feelings of loneliness, smoking addiction, alcoholism, academic grade, ideation of suicide, exercise, and having a part-time job; (2) 25 social and economic factors: father’s education level, mother’s education level, father’s employment, mother’s employment, monthly household income per capita (yuan), single parent, ability to deal with interpersonal relationships, school loans, strained relationship with classmates and friends, strained relationship with teacher, strained relationship with parents, uncomfortable relationship with people of the opposite sex, bad employer, being disappointed in love, being discriminated against, being criticized or misunderstood, having sincere friends, death of relatives, acute and serious illness of relatives, emergency of a family member, lack of psychological guidance, family medical history, number of home moves, parental rearing behaviour, and parental attention level; and (3) 2 environmental factors: social support and unsatisfaction with social phenomenon.

### Quality of the included articles

The determinants of depression or anxiety were analysed via a meta-analysis and content analysis. The average score of the included articles was 6.14 out of 7 according to the modified Newcastle-Ottawa scale (shown in Table 2). All studies were of medium and high quality. Fifteen articles did not meet the standard of representativeness of the sample (sample size ≥1000 participants). Three articles did not report the non-respondents or reported a response rate of less than 80%. All articles met the other conditions. The quality assessment for this systematic review is shown in Table 2.

### Analysis of depression

In the 21 identified articles, 15 reported the prevalence of depression. Based on the 15 studies, the prevalence of depression ranged from 13.10 to 76.21%, as shown in Table 3. The mean prevalence of depression was 32.74%. However, different studies used different assessment tools. For example, the Beck Depression Inventory (BDI) defined a score of > 5 out of 63 to indicate depression. The Centre for Epidemiologic Studies Depression Scale (CES-D) defined a score of > 15 out of 60 to indicate depression. The Self-rating Depression Scale (SDS) defined a score of > 40 out of 80 to indicate depression.

Amongst all the determinants evaluated, the depression analysis extracted 31 determinants, including 24 individual factors, six social and economic factors, and one environmental factor.

Six of the individual factor determinants were included in the meta-analyses whereas all the extracted determinants were included in the descriptive analysis. Figure 2 shows the meta-analysis results of the depression-related individual factors. Gender (male vs female, OR: 1.17, 95% CI: 1.10–1.24), grade level (low grade vs high grade, OR: 0.77, 95% CI: 0.73–0.81, *P* < 0.001), residence (rural vs urban: OR: 0.74, 95% CI: 0.68–0.81, *P* < 0.001), and satisfaction with current major (satisfied vs unsatisfied: OR: 0.48, 95% CI: 0.40–0.56, *P* < 0.001) were significantly associated with depression in medical students. Ethnicity was not significantly associated with depression in medical students. The studies found that male students, higher grade level students, and students who were unsatisfied with their current majors were more depressed than their peers [62, 64, 74].

Medical students who had an increased odds of having depression were those who had siblings [63, 74] and were from rural areas [63]. Higher depressive symptoms were also reported in medical students with smoking [64, 68] and drinking [64, 68] habits, poor adaptability [62], sleep deprivation [64, 77], a longer length of schooling [64], and in those who had been hospitalized or received medication for 1 week or more in the last 4 weeks [64], were in a lower academic grade level [72], had a bad self-evaluation of mental health [35, 72], had a higher frequency of participating in association activities [35], had a higher interest [35, 78] and satisfaction [70] with their current major, had a pessimistic anticipation of their future career [35], had greater academic pressure [35, 68, 78], and experienced the ideation of suicide [72].

When evaluating school aspects, the students in traditional Chinese medical schools or comprehensive schools [64] experienced higher levels of depression symptoms [66]. Regarding current major, pharmacy students [34, 72] and nursing students [34] were more depressed than their peers. Previous studies have not reached the same conclusions regarding the above issues.

One of the social and economic factor determinants was analysed in the meta-analysis whereas all the extracted determinants were included in the descriptive analysis. Figure 2 shows the meta-analysis results of the depression-related social and economic factors. The monthly household income per capita (below 2000 vs above 2000: OR: 1.34, 95% CI: 1.24–1.45, *P* < 0.001) was significantly associated with depression in medical students. Medical students who experienced higher depressive symptoms were those who had a low monthly family income per capita [64, 70, 72], parents with a poor educational background [64], a strained relationship with classmates [35, 72], a broken family [68, 70], financial debts [35], and a family medical history [68].

In the assessment of environmental factors, the social support factor negatively affected depression [68, 70, 75].

### Analysis of anxiety

In the 21 identified studies, 11 reported on the prevalence of anxiety. Based on the 11 articles, the prevalence of anxiety ranged from 8.54 to 88.30%, as shown in Table 2. The mean prevalence of anxiety was 27.22%. However, 6 different assessment tools were used in different articles. For example, the Beck Anxiety Inventory (BAI) defined a score of > 5 out of 63 to indicate anxiety. The Hamilton Anxiety Scale (HAMA) defined a score of > 14 out of 56 to indicate anxiety. The Self-rating Anxiety Scale (SAS) defined a score of > 40 out of 80 to indicate anxiety.

Amongst all the determinants evaluated, the anxiety analysis identified 20 relevant determinants, including 13 individual factors, six social and economic factors, and one environmental factor.

**Fig. 3:**
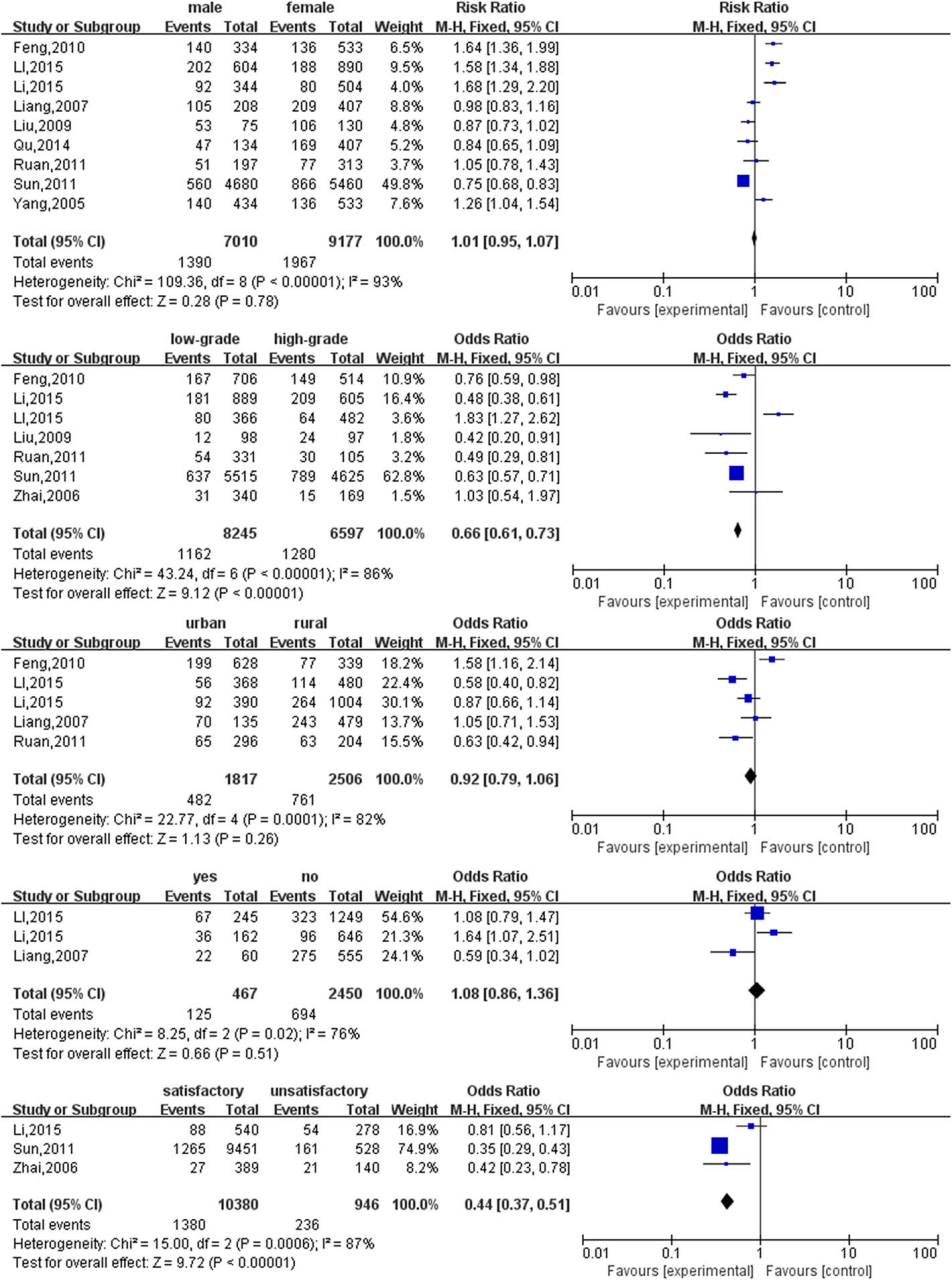
Forest plots of anxiety-related factors

Five individual factor determinants were included in the meta-analysis whereas all the extracted determinants were included in the descriptive analysis. Figure 3 shows the meta-analysis results of the anxiety-related individual factors. After performing the meta-analysis, grade level (low grade vs high grade, OR: 0.66, 95% CI: 0.61–0.73, *P* < 0.00001) and satisfaction with current major (satisfied vs unsatisfied: OR: 0.44, 95% CI: 0.37–0.51, *P* < 0.001) were significantly associated with anxiety. Gender, house registry, and being an only child were not significantly associated with anxiety. According to the meta-analysis, the higher grade level students and students who were unsatisfied with their current major were more anxious than their peers.

Medical students who were ethnic minorities [71], lived in rural areas [65, 69, 71], and had siblings [69, 76] tended to experience anxiety symptoms. Medical students who had poor interpersonal relationships [65], higher academic pressure [65], lower interest and satisfaction with current major [67, 69, 70], poor self-evaluation [69], a poor anticipation of future careers, and sleep deprivation [77] had increased odds of having anxiety.

Regarding current major, the Chinese and Western clinical medicine or nursing students were more depressed than their peers [75, 76]. Other studies have not reached the same conclusion on this issue [86].

Social and economic factors: anxiety was more likely to affect medical students who had a lower monthly household income per capita (Yuan) [34, 70], parents with a poor educational background [65], on-the-job parents [69], a broken family [70], and had experienced coincidental bad events [67].

Environmental factors: the students who had more social support tended to be less anxious [67, 70].

## Discussion

Our study conducted a systematic review of the prevalence and determinants of depression and anxiety amongst medical students in China. Fifteen studies reported on the prevalence of depression amongst medical students whereas 11 studies reported on the prevalence of anxiety.

The prevalence of depression and anxiety were consistent with related studies conducted in other countries. According to a previous study, the overall global prevalence of depression or depressive symptoms amongst medical students was 27.2% [87]. The prevalence of depression and anxiety outside the North American region was 7.7–65.5% and 6.0–66.5%, respectively [87, 88]. In Turkey, depression and anxiety prevalence rates of 39.0 and 35.8% were reported, respectively [74]. In Egypt, high frequencies of depression (65%), anxiety (73%) and stress (59.9%) were reported. In Nepal, the overall prevalence of depression was 29.9%, and the prevalence of anxiety was 41.1%. The status of anxiety was more severe than the status of depression. A different research scope could explain the discrepancies found in different areas [8].

This study also identified 57 determinants related to depression or anxiety, including 30 individual factors, 25 social and economic factors, and two environmental factors.

In terms of individual factors, first, according to the other studies, the gender differences are changing over time in China; older research tended to report that female students were more depressed or anxious than male students, whereas recent research tended to report the opposite conclusion. This finding is thought to be a result of the higher expectations and responsibilities of male students [62, 77]. However, in other countries, female students normally displayed higher depressive and anxious symptoms [18, 89]. Conversely, studies in some countries reported no gender differences [90]. In China, people still held the perception that men primarily responsible for the “outside part” in family functioning such as earning money whereas women are responsible for the “inside part”, such as running the household [91]. Second, the most depressive and anxious grade levels were sophomore and senior students. According to the studies, sophomore students experienced higher academic pressure. Conversely, facing employment pressure, senior students were in a period of transition and feared the future uncertainty. In other studies, freshmen and senior students reported the highest rates of depression and anxiety [89, 92]. Third, students with siblings were more depressed and anxious than only children, partly because the latter received more attention from their parents. A previous study reported the same results [93]. Fourth, rural students experienced higher depressive and anxious conditions. Rural students were faced with higher financial stress and family burdens. Medical students from a lower socioeconomic status were more depressed and anxious than their counterparts [64], which suggests that students from low-income families should be given more attention than their peers [94]. Fifth, the degree of dissatisfaction with education was associated with depression and anxiety [95]. In studies from India and Brazil, the low frequency of exercise and high frequency of taking medicines were potential risk factors of depressive and anxious symptoms [86, 96]. In North America, the degrees of depression and anxiety were associated with cigarette addiction, alcoholism, and sleeping deprivation [96]. Medical students in comprehensive colleges were more depressed and anxious than those from medical universities, according to research in Nepal. In Malaysia, the prevalence of depression and anxiety was higher amongst students who had difficulty dealing with interpersonal relationships and who had a family history of depression and anxiety [18, 20, 89, 90, 92].

Social and economic factors included relationships with family members, classmates, teachers, and companions. The fathers’ and mothers’ educational backgrounds and employment were related to depressive and anxious symptoms. Parents with a higher degree reflected lower depressive and anxious symptoms [97]. Unemployed parents increased the stress of medical students, particularly for students with an unemployed father. The fathers were expected to take up the role of breadwinner whereas the mothers were responsible for the household chores [30]. There was an increasing trend in the prevalence of depressive and anxious symptoms associated with the monthly income per capita of the family. In studies from India, Brazil, Nepal, America, and Malaysia, a higher prevalence of depressive and anxious symptoms was reported amongst students with family problems and a family history of depression and anxiety [18, 90].

In the analysis of environmental factors, the results suggested that social support played an important role in moderating depressive and anxious symptoms [98].

One strength of this review was that the analysis of the prevalence of depression and anxiety was based on a large sample size of 21 articles and 35,160 individuals. Another strength was the comprehensive analyses of the determinants, particularly the meta-analysis and content analysis. Fifty-nine determinants were extracted from the 21 studies. Thirty-one determinants were significantly associated with depression, and 20 were significantly associated with anxiety. However, some limitations exist in this systematic review. There was significant heterogeneity amongst the individual studies when performing the meta-analysis. To address the problem, more studies should be included by changing search strategy Furthermore, it was difficult to integrate the prevalence of depression and anxiety of included articles, which calculated the values with different assessment tools. The assessment tools had distinct standard, so the integrated results in our article only relatively revealed the true value. In future, the results from different assessment tools should be integrated respectively.

## Conclusions

This systematic review and meta-analysis highlight the problem of depression and anxiety amongst medical students and the related determinants. The findings shed light on taking measures to create potential solutions to diminish mental health diseases. From the perspective of health authorities, the government should invest more money into medical students to satisfy their basic daily needs, especially for the students with financial problem. Early detection and prevention programmes play an important role in alleviating mental problems. From the perspective of medical schools in China, a lot of depressive and anxious medical students were influenced by school-related factors. Schools should strengthen mental support in college, including popularizing psychological knowledge, providing mental health-related courses and lectures, and creating counselling centres. From an individual perspective, students tend to deny being ill because of negative attitudes regarding mental health in society [99]. Therefore, students must confront their mental health problems rather than avoid them. Furthermore, additional studies that explore the environmental factors in the Chinese context are necessary.

## Additional file

Additional file 1: The Search Strategy & The extracted determinants & The distribution of determinants & The Extracted Determinants & The Significant Results. (DOCX 944 kb)

## Data Availability

Data sharing is not applicable to this article as no datasets were generated or analyzed during the current study. All databases used in this study are open to the public.

## Abbreviations

ADHS: Adult Dispositional Hope Scale
APGAR: Family APGAR Index
ASLEC: Adolescent Self-Rating Life Events Check List
BAI: Beck Anxiety Inventory
BDI: Beck Depression Inventory
BHS: Beck Hopeless Scale
CBM: China Biology Medicine disc
CCSAS: China College Student Adjustment Scale
CES-D: Center for Epidemiologic Studies Depression Scale
CMB: China Medical Board
CNKI: China National Knowledge Internet Database
CSQ: Coping Style Questionnaire
EF: Environmental factors
EPQ: Eysenck Personality Questionnaire
GRADE: The Grading of Recommendations: Assessment, Development, and Evaluation
HAMA: Hamilton anxiety scale
IF: Individual factors
LES: Life Events Scale
LOT-R: Life Orientation Test-Revised
OR: Odds Ratio
PRISMA: Preferred Reporting Items for Systematic Reviews and Meta-Analyses
PSQI: The Pittsburgh Sleep Quality Index
PSSS: Perceived Social Support Scale
RS-14: Resilience Scale-14
SAS: The Self-rating Anxiety Scale
SCL-90: Symptom Checklist 90
SDH: Social determinants of health
SDS: The self-rating Depression Scale
SEF: Social and economic factors
SF-36: The Medical Outcomes Study 36-Item Short-form Health Survey
SLS: Student-life Stress Inventory
SSRS: Social Support Rating Scale
TX: Texas
UK: The United Kingdom
USA: The United State
WHO: World Health Organization
