# Supplementary material for: A systematic review of depression and anxiety in medical students in China
Source: BMC Med Educ. 2019 Sep 2;19:327. doi: 10.1186/s12909-019-1744-2 (PMC6721355; doi:10.1186/s12909-019-1744-2)

**Appendix 1 The Search Strategy**

**Keyword**

China/Chinese

Medical students/ Trainee doctor/undergraduate medical students/student doctor

Depression/Major depressive disorder/Depressive disorder/anxiety

中国、中国的

医学生、医院实习生、医学专业学生

焦虑、抑郁

**Search strategy**

**Database 1: CAJD (CNKI)**

(SU=('医学生'+'医院实习生'+'医学专业学生')AND SU=('抑郁'+'焦虑'))

**Database 2: CSPD (WANFANG Data)**

(题名或关键词:(医学生)+题名或关键词:(医院实习生)+题名或关键词:(医院专业学生))and(题名或关键词:(抑郁)+题名或关键词:(焦虑))

**Database 3: CBM**

(((("医学生抑郁"[标题] OR "医学生焦虑"[标题]) OR "医院实习生抑郁"[标题]) OR "医院实习生焦虑"[标题]) OR "医院专业学生抑郁"[标题]) OR "医院专业学生焦虑"[标题]Database

**Database4: PubMed (NCBI)**

| Sequence | Query |
| --- | --- |
| #1 | Search (China[Title/Abstract]) OR China[MeSH Terms] |
| #2 | Search (Chinese[Title/Abstract]) OR Chinese[MeSH Terms] |
| #3 | Search (Medical students[Title/Abstract]) OR Medical students[MeSH Terms] |
| #4 | Search (Trainee doctor[MeSH Terms]) OR Trainee doctor[Title/Abstract] |
| #5 | Search (undergraduate medical students[Title/Abstract]) OR undergraduate medical students[MeSH Terms] |
| #6 | Search (student doctor[Title/Abstract]) OR student doctor[MeSH Terms] |
| #7 | Search (Depression[Title/Abstract]) OR Depression[MeSH Terms] |
| #8 | Search (Major depressive disorder[Title/Abstract]) OR Major depressive disorder[MeSH Terms] |
| #9 | Search (Depressive disorder[Title/Abstract]) OR Depressive disorder[MeSH Terms] |
| #10 | Search (anxiety[Title/Abstract]) OR anxiety[MeSH Terms] |
| #11 | #1 OR #2  Search (((China[Title/Abstract]) OR China[MeSH Terms])) OR ((Chinese [Title/Abstract]) OR Chinese[MeSH Terms]) |
| #12 | #3 OR #4 OR #5 OR #6  Search (((((Medical students[Title/Abstract]) OR Medical students[MeSH Terms])) OR ((Trainee doctor[MeSH Terms]) OR Trainee doctor[Title/Abstract])) OR ((undergraduate medical students[Title/Abstract]) OR undergraduate medical students[MeSH Terms])) OR ((student doctor[Title/Abstract]) OR student doctor[MeSH Terms]) |
| #13 | #7 OR #8 OR #9 OR #10  Search (((((Depression[Title/Abstract]) OR Depression[MeSH Terms])) OR ((Major depressive disorder[Title/Abstract]) OR Major depressive disorder[MeSH Terms])) OR ((Depressive disorder[Title/Abstract]) OR Depressive disorder[MeSH Terms])) OR ((anxiety[Title/Abstract]) OR anxiety[MeSH Terms]) |
| #14 | #11 AND #12 AND #13  Search ((((((China[Title/Abstract]) OR China[MeSH Terms])) OR ((Chinese[Title/Abstract]) OR Chinese[MeSH Terms]))) AND ((((((Medical students[Title/Abstract]) OR Medical students[MeSH Terms])) OR ((Trainee doctor[MeSH Terms]) OR Trainee doctor[Title/Abstract])) OR ((undergraduate medical students[Title/Abstract]) OR undergraduate medical students[MeSH Terms])) OR ((student doctor[Title/Abstract]) OR student doctor[MeSH Terms]))) AND ((((((Depression[Title/Abstract]) OR Depression[MeSH Terms])) OR ((Major depressive disorder[Title/Abstract]) OR Major depressive disorder[MeSH Terms])) OR ((Depressive disorder[Title/Abstract]) OR Depressive disorder[MeSH Terms])) OR ((anxiety[Title/Abstract]) OR anxiety[MeSH Terms])) |

**Database5: EMBASE**

| No. | Query |
| --- | --- |
| #1 | 'china'/exp |
| #2 | 'china':ti,ab |
| #3 | #1 OR #2 |
| #4 | 'medical students'/exp |
| #5 | 'medical students':ti,ab |
| #6 | 'trainee doctor':ti,ab |
| #7 | 'undergraduate medical students':ti,ab |
| #8 | 'student doctor':ti,ab |
| #9 | #4 OR #5 OR #6 OR #7 OR #8 |
| #10 | 'depression'/exp |
| #11 | 'anxiety'/exp |
| #12 | 'depression':ti,ab |
| #13 | 'anxiety':ti,ab |
| #14 | 'major depressive disorder':ti,ab |
| #15 | 'depressive disorder':ti,ab |
| #16 | #10 OR #11 OR #12 OR #13 OR #14 OR #15 |
| #17 | #3 AND #9 AND #16 |

**Database6: Cochrane library**

| ID | Search |
| --- | --- |
| #1 | China’:ti,ab,kw (Word variations have been searched) |
| #2 | Chinese’:ti,ab,kw(Word variations have been searched) |
| #3 | #1 or #2 |
| #4 | Medical students: ti,ab,kw (Word variations have been searched) |
| #5 | Trainee doctor: ti,ab,kw (Word variations have been searched) |
| #6 | student doctor: ti,ab,kw (Word variations have been searched) |
| #7 | undergraduate medical students: ti,ab,kw (Word variations have been searched) |
| #8 | #4 or #5 or #6 or #7 |
| #9 | Depression: ti,ab,kw (Word variations have been searched) |
| #10 | Major depressive disorder: ti,ab,kw (Word variations have been searched) |
| #11 | Depressive disorder: ti,ab,kw (Word variations have been searched) |
| #12 | anxiety: ti,ab,kw (Word variations have been searched) |
| #13 | #9 or #10 or #11 or #12 |

**Appendix 2 The Extracted determinants**

**57 determinants**

**Depression and anxiety**

| **Group** | **Category** | **Determinant** |
| --- | --- | --- |
| A | Individual factors | A1-gender, A2-grade, A3-age, A4-ethnicity, A5-residence, A6-current major, A7-type of school, A8-school location, A9-learning stage, A10-length of schooling, A11- number of siblings, A12-rating of school, A13-satisfication with current major, A14-attitude towards future career, A15-academic pressure, A16-clinical internship, A17-retaking college entrance examination as a senior, A18-personal character score, A19-adaptability factor score,  A20-interest of current major, A21-satisfacation with school life, A22-satisifacation with accommodation, A23-sleeping conditions, A24-feelings of loneliness, A25-smoking addiction, A26-alcoholism, A27- academic grade, A28-ideation of suicide, A29-exercise, A30-taking part time job |
| B | Social and economic factors | B1-father’s education level, B2-mother’s education level, B3-father’s employment, B4-mother’s employment, B5- monthly household income per capita (Yuan), B6-Single parent, B7-ability to deal with interpersonal relationship; B8-school loan,  B9-strained relationship with classmates and friends, B10-strained relationship with teacher, B11-strained relationship with parents, B12-uncomfortabled relationship with opposite sex people, B13-bad employer B14-being disappointed in love, B15-being discriminated, B16-being criticized or understood, B17-having sincere friends, B18-death of relatives, B19-acute and serious illness of relatives, B20-emergency of family number, B21-lacking of psychological guidance, B22-family medical history, B23-home moving times, B24-parental rearing behavior, B25-parental attention level |
| C | environmental factors | C1-social support score;  C2-unsatisfaction of social phenomenon |

*classification standard

Individual factors (IF) (including biological factors, psychological factors, and behavioral factors), social and economic determinants (SED) and risk factors at environmental factors (EF) (including inequality, racism, discrimination, refugee, war and immigrants)

**Appendix 3 The Distribution of Determinants**

**Distribution of determinants in 21 included studies (1 = Yes, space = No)**

| **Determinants** | **Included studies** | | | | | | | | | | | | | | | | | | | | | **total** |
| --- | --- | --- | --- | --- | --- | --- | --- | --- | --- | --- | --- | --- | --- | --- | --- | --- | --- | --- | --- | --- | --- | --- |
|  | **1** | **2** | **3** | **4** | **5** | **6** | **7** | **8** | **9** | **10** | **11** | **12** | **13** | **14** | **15** | **16** | **17** | **18** | **19** | **20** | **21** |  |
| **A1** | **1** | **1** | **1** | **1** | **1** | **1** | **1** |  | **1** | **1** | **1** | **1** | **1** | **1** | **1** | **1** | **1** | **1** | **1** | **1** | **1** | **20** |
| **A2** | **1** | **1** | **1** |  | **1** |  |  |  | **1** | **1** | **1** | **1** |  | **1** |  |  | **1** |  | **1** | **1** | **1** | **13** |
| **A3** |  |  | **1** |  |  |  | **1** |  |  |  | **1** |  |  |  |  |  | **1** |  | **1** |  |  | **5** |
| **A4** |  |  | **1** |  | **1** | **1** | **1** |  | **1** | **1** | **1** |  |  | **1** |  | **1** | **1** | **1** | **1** | **1** | **1** | **14** |
| **A5** |  |  | **1** | **1** |  | **1** |  |  |  |  |  |  | **1** |  |  |  | **1** |  | **1** |  |  | **6** |
| **A6** |  |  |  |  |  |  | **1** |  |  |  |  |  |  |  |  |  |  |  |  |  |  | **1** |
| **A7** |  | **1** |  |  | **1** |  | **1** |  |  |  |  | **1** |  |  | **1** | **1** |  |  |  | **1** |  | **7** |
| **A8** |  |  | **1** |  |  |  | **1** |  |  |  |  |  |  |  |  |  |  |  |  |  |  | **2** |
| **A9** |  |  |  |  |  |  |  |  |  |  |  | **1** | **1** |  |  |  |  |  |  |  |  | **2** |
| **A10** | **1** |  |  |  |  | **1** | **1** |  |  |  |  |  |  |  |  |  |  |  |  |  |  | **3** |
| **A11** |  |  |  |  |  |  |  |  |  |  |  |  |  |  |  | **1** |  |  |  |  |  | **1** |
| **A12** |  |  |  |  |  |  |  |  |  |  |  |  |  |  | **1** |  |  |  |  |  |  | **1** |
| **A13** |  |  | **1** |  | **1** |  |  |  | **1** | **1** | **1** |  |  |  |  | **1** |  |  | **1** |  |  | **7** |
| **A14** |  |  |  |  | **1** |  | **1** |  |  |  | **1** |  |  |  |  |  |  |  |  |  |  | **3** |
| **A15** |  |  |  |  |  |  |  |  |  |  | **1** | **1** |  |  |  |  |  |  |  |  |  | **2** |
| **A16** |  |  |  |  |  |  |  |  |  |  |  |  | **1** |  |  |  | **1** |  |  |  |  | **2** |
| **A17** |  |  |  |  |  |  |  |  | **1** |  |  |  |  |  |  | **1** |  | **1** |  |  |  | **3** |
| **A18** |  |  |  |  |  |  |  |  |  |  |  |  |  |  | **1** |  |  |  |  |  |  | **1** |
| **A19** |  |  |  |  |  | **1** | **1** |  |  |  |  |  |  |  |  |  |  |  |  |  |  | **2** |
| **A20** |  |  |  |  |  |  |  | **1** | **1** |  |  |  |  |  |  |  |  |  |  |  |  | **2** |
| **A21** |  |  |  |  |  |  |  |  | **1** |  | **1** |  |  |  | **1** |  |  |  |  |  |  | **3** |
| **A22** |  |  |  |  |  |  |  |  |  |  |  |  | **1** | **1** |  |  |  |  |  |  |  | **2** |
| **A23** |  |  |  |  | **1** |  |  |  |  |  |  |  |  |  |  | **1** |  |  |  |  |  | **2** |
| **A24** |  |  |  |  |  |  |  |  |  |  |  |  |  |  | **1** |  |  |  |  |  |  | **1** |
| **A25** |  |  |  |  |  |  | **1** |  |  | **1** |  |  |  |  |  |  |  |  |  |  |  | **2** |
| **A26** |  |  |  |  |  |  |  | **1** |  |  |  |  |  |  |  |  |  |  |  |  |  | **1** |
| **A27** |  |  |  |  |  |  |  | **1** |  |  |  |  |  |  |  |  |  | **1** |  |  |  | **2** |
| **A28** |  |  |  |  |  |  | **1** |  |  |  |  |  |  |  |  |  |  | **1** |  |  |  | **2** |
| **A29** |  |  |  |  |  |  | **1** |  |  |  |  |  |  |  |  |  |  |  |  |  |  | **1** |
| **A30** | **1** |  |  |  |  |  |  |  |  |  |  |  |  |  |  |  |  |  |  |  |  | **1** |
| **B1** |  |  |  |  |  | **1** | **1** |  | **1** |  | **1** |  |  |  |  | **1** |  |  |  |  | **1** | **6** |
| **B2** |  |  |  |  |  | **1** | **1** |  | **1** |  | **1** |  |  |  |  | **1** |  |  |  |  | **1** | **6** |
| **B3** |  |  |  |  |  |  |  |  |  |  |  |  | **1** |  |  |  |  |  |  |  | **1** | **2** |
| **B4** |  |  |  |  |  |  |  |  |  |  |  |  | **1** |  |  |  |  |  |  |  | **1** | **2** |
| **B5** |  |  |  |  |  |  |  |  |  |  |  |  |  |  | **1** |  |  |  |  |  |  | **1** |
| **B6** |  |  |  |  |  |  |  |  |  |  |  |  |  |  |  | **1** |  |  | **1** |  |  | **2** |
| **B7** |  |  |  |  |  |  |  |  | **1** |  |  | **1** |  |  |  |  | **1** | **1** |  |  |  | **4** |
| **B8** |  |  |  |  |  |  |  |  |  |  | **1** | **1** |  |  |  |  |  |  |  |  |  | **2** |
| **B9** |  |  |  |  |  |  |  |  | **1** | **1** |  |  |  |  |  |  |  |  |  |  |  | **2** |
| **B10** |  |  |  |  |  | **1** | **1** |  |  |  |  |  |  |  |  |  |  |  |  |  |  | **2** |
| **B11** |  |  |  |  |  |  |  |  |  |  |  | **1** |  |  |  |  | **1** |  |  |  |  | **2** |
| **B12** |  |  |  |  |  |  |  |  |  |  |  |  |  |  |  |  |  | **1** |  |  |  | **1** |
| **B13** |  |  |  |  |  |  |  |  |  |  |  |  |  |  |  |  |  |  | **1** |  |  | **1** |
| **B14** |  |  |  |  |  |  |  |  |  | **1** |  |  |  |  |  |  | **1** |  |  |  |  | **2** |
| **B15** |  |  |  |  |  |  |  |  | **1** |  | **1** |  |  |  |  |  |  |  |  |  |  | **2** |
| **B16** |  |  |  |  |  |  |  |  |  |  |  |  |  |  | **1** |  |  |  |  |  |  | **1** |
| **B17** |  |  |  |  |  |  |  |  |  |  | **1** | **1** |  |  |  |  |  |  |  |  |  | **2** |
| **B18** |  |  |  |  |  |  |  |  |  |  |  |  |  |  |  |  |  |  |  | **1** |  | **1** |
| **B19** |  |  |  |  |  |  |  |  |  |  |  |  |  |  |  |  | **1** |  |  |  |  | **1** |
| **B20** |  |  |  |  |  |  | **1** |  |  |  |  |  |  |  |  |  |  |  |  |  |  | **1** |
| **B21** |  |  |  |  |  |  |  |  |  |  | **1** |  |  |  |  |  |  |  |  |  |  | **1** |
| **B22** |  |  |  |  |  |  |  |  |  |  |  |  |  |  |  |  |  | **1** |  |  |  | **1** |
| **B23** |  |  |  |  |  |  |  |  |  |  |  |  |  |  |  | **1** |  |  |  |  |  | **1** |
| **B24** |  |  |  |  |  |  |  |  |  |  |  |  |  |  |  | **1** |  |  |  |  |  | **1** |
| **B25** |  |  |  |  |  |  |  |  |  |  |  |  |  |  |  | **1** |  |  |  |  |  | **1** |
| **C1** | **1** |  |  |  |  |  |  |  |  |  |  |  |  |  |  |  |  | **1** |  |  |  | **2** |
| **C2** |  |  |  |  |  |  |  |  |  |  |  |  |  |  |  |  |  | **1** |  |  |  | **1** |
| **Total** | **5** | **3** | **7** | **2** | **7** | **8** | **16** | **3** | **12** | **7** | **14** | **9** | **7** | **4** | **8** | **13** | **10** | **10** | **8** | **5** | **7** | **165** |

**Appendix 4 The Extracted Determinants**

**Data extraction according to each determinant**

**DEPRESSION AND NON-DEPRESSION**

A1-gender

| Author | Group | Depression | non-depression | Results |
| --- | --- | --- | --- | --- |
| Lin, 2018 | Male  Female | 63  57 | 339  454 | Significance different  (χ^2^ test=4,53, p＜0.05) |
| Li, 2017 | Male  Female | 451  1040 | 357  902 | Significance different  (χ^2^ test=3.32, p＜0.0685) |
| Pan, 2016 | Male  Female  Missing | 845  881 | 2570  4613  101 | Significance different  (χ^2^ value is not stated, p＜0.001)  Male (95%CI: 1.39-1.79  OR=1.58, adjusted) |
| Qu, 2014 | Male  Female | 42  121 | 88  286 | No significance different  (χ^2^ =0.29, p=0.59) |
| Guo, 2014 | Male  Female | 74  162 | 69  149 | No significance different  (χ^2^ test=0.25, p=0.802) |
| Ren, 2013 | Male  Female | 36  32 | 106  110 | No significance different  (χ^2^value is not stated, p=0.781) |
| Sun, 2011 | Male  female | 738  961 | 4680  5460 | No significance different  (χ^2^ test=7.20, p＞0.05) |
| Shang, 2009 | Male  Female | 192  402 | 313  603 | No significance different  (χ^2^ test=0.53, p＞0.05) |
| He, 2009 | Male  Female | 166  253 | 437  792 | No significance different  (χ^2^ =2.22, p=0.14) |
| Yang, 2005 | Male  Female | 40  39 | 214  187 | No significance different  (χ^2^ =0.2097, p=0.9013) |
| Zhou, 2003 | Male  Female | 31  40 | 88  88 | Significance different  (χ^2^ =13.39, p＜0.01) |
| Yu, 2001 | Male  Female | 182  66 | 180  82 | Significance different  (χ^2^ =0.90, p＞0.05) |

A2-grade

| Author | Group | Depression | non-depression | Results |
| --- | --- | --- | --- | --- |
| Lin, 2018 | Freshmen  Sophomore (the second grade)  Sophomore (the third grade)  Junior  senior | 27  33  27  26  7 | 159  227  152  195  60 | Significance different  (χ^2^ test =4.34, p＜0.05) |
| Li, 2017 | Freshmen  Sophomore  Junior  Senior | 354  352  386  399 | 352  303  356  248 | Significance different  (χ^2^ test=21.73, p＜0.0001) |
| Sun, 2011 | Freshmen  Sophomore | 718  981 | 5515  4625 | Significance different  (χ^2^ test=129.31, p＜0.001)  Freshmen (95%CI: 0.72-0.92, OR=0.81) |
| He, 2009 | Freshmen  Sophomore (the second grade)  Sophomore (the third grade)  Junior  senior | 114  136  94  46  28 | 330  374  228  157  143 | Significance different  (χ^2^ test=11.07, p=0.03) |
| Zhai, 2006 | Freshmen  Sophomore  Junior  Senior | 27  50  32  5 | 149  114  92  40 | Significance different  (χ^2^ test=15.13, p＜0.05) |
| Yu,2001 | Freshmen  Sophomore  Junior  senior | 81  58  54  54 | 82  58  53  69 | Significance different  (F test=0.9105, p＞0.05) |

A3-age

| Author | Group | Depression | non-depression | Results |
| --- | --- | --- | --- | --- |
| Pan, 2016 | 15-19  20-22  23-28  Missing | 289  1085  311 | 1669  4535  894  227 | Significance different  (χ^2^ value is not stated, p＜0.001)  20-22 (95%CI: 0.76-1.14, OR=0.93, adjusted),  23-28 (95%CI: 0.91-1.58, OR=1.20, adjusted) |
| Sun, 2011 | 15-19  20-25 | 645  1054 | 4640  5500 | Significance different  (χ^2^test=50.06, p＜0.001) |

A4-ethnicity

| Author | Group | Depression | non-depression | Results |  |
| --- | --- | --- | --- | --- | --- |
| Li，2017 | Han nationality  Minority nationality | 1262  229 | 1116  143 | Significance different  (χ^2^ test=15.23, p＜0.0001) |  |
| Pan, 2016 | Han nationality  Minority nationality  Missing | 1546  147 | 6450  624  243 | No significance different  (χ^2^ is not stated, p=0.878) |  |
| He, 2009 | Yi nationality  Bai nationality  Hui nationality  Dai nationality  Zhuang nationality  Hani nationality  Miao nationality  others | 117  58  62  35  33  21  8  84 | 379  236  154  88  70  56  31  222 | No significance different  (χ^2^ =11.42, p=0.12) | |

A5-house registry

| Author | Group | Depression | non-depression | Results |
| --- | --- | --- | --- | --- |
| Li, 2017 | Urban  Rural | 511  980 | 541  718 | Significance different  (χ^2^ test=20.26, p＜0.0000) |
| Pan, 2016 | Urban  Rural  Missing | 587  1060 | 2971  3970  422 | Significance different  (χ^2^ value is not stated, p＜0.001)  Rural (95%CI: 0.87-1.18, OR=1.01, adjusted) |
| Zhou, 2003 | Urban  Town  County  Rural | 28  9  7  17 | 39  19  37  81 | No significance different  (χ^2^ test=0.22, p＞0.05) |

A6-current major

| Author | Group | Depression | non-depression | Results |
| --- | --- | --- | --- | --- |
| Pan, 2016 | Clinical or dental  Non-clinical or –dental | 809  893 | 3302  3908  98 | No significance different  (χ^2^ value is not stated, p=0.139) |
| Guo, 2014 | Nursing major  Preventive medicine major  Clinical major  Pharmacy  Chinese medicine | 43  63  74  43  23 | 15  55  96  38  23 | Significance different  (χ^2^ test=19.84, p＜0.001) |

A7-school

| Author | Group | Depression | non-depression | Results |
| --- | --- | --- | --- | --- |
| Pan, 2016 | Medical  Comprehensive | 888  863 | 4128  3131 | Significance different  (χ^2^ value is not stated, p＜0.001)  Comprehensive (95%CI: 1.43-2.57, OR=1.21) |
| Sun, 2011 | University 1  University 2  University 3 | 413  804  482 | 2904  4261  2975 | Significance different  (χ^2^test=31.51, p＜0.001)  College 1 (95%CI: 0.59-0.78, OR=0.68)  College 2 (95%CI: 0.67-0.87, OR=0.77) |

A8-school location

| Author | Group | Depression | non-depression | Results |
| --- | --- | --- | --- | --- |
| Li, 2017 | Eastern area  Middle area  Western area | 336  436  719 | 331  406  522 | Significance different  (χ^2^ test=17.98, p＜0.0001) |
| Pan, 2016 | East  Central  West | 349  890  512 | 1834  3602  1823 | Significance different  (χ^2^value is not stated, p＜0.001) |

A9-educational background

| Author | Group | Depression | Non-depression | Results |
| --- | --- | --- | --- | --- |
| He, 2009 | Graduate student  Undergraduate student  Junior college education | 2  356  61 | 14  1027  191 | No significance different  (χ^2^test=1.91, p=0.39) |

A10-year of study

| Author | Group | Depression | non-depression | Results |
| --- | --- | --- | --- | --- |
| Pan, 2016 | 1-2  3  4-5 | 403  622  716 | 2284  2365  2549  71 | Significance different  (χ^2^ value is not stated, p＜0.001)  3 (95%CI: 1.08-1.60, OR=1.32)  4-5 (95%CI: 0.92-1.41, OR=1.12) |

A11-no sibling

| Author | Group | Depression | non-depression | Results |
| --- | --- | --- | --- | --- |
| Li, 2017 | Having sibling  No sibling | 1044  447 | 810  449 | Significance different  (χ^2^ test=10.11, p＜0.0015) |
| He, 2009 | Having sibling  No sibling | 294  125 | 894  337 | Significance different  (χ^2^ test=4.58, p=0.03) |

A12-rating of school

| Author | Group | | Depression | non-depression | Results |
| --- | --- | --- | --- | --- | --- |
| Pan, 2016 | | First-class  Non first-class | 951  800 | 3963  3296 | No significance different  (χ^2^ value is not stated, p=0.846) |

A13-satisfaction with current major

| Author | Group | Depression | non-depression | Results |
| --- | --- | --- | --- | --- |
| Sun, 2011 | Very satisfied  Satisfied  Non-satisfied | 411  854  161 | 3302  4884  528 | Significance different  (χ^2^ =86.34, p＜0.01)  Very satisfied (95%CI: 0.29-0.43, OR=0.36)  Satisfied (95%CI: 0.48-0.68, OR=0.57) |
| Zhai, 2006 | Satisfied  Relatively satisfied  Relatively unsatisfied  Unsatisfied | 39  29  34  14 | 169  152  77  15 | Significance different  (χ^2^ =21.01, p＜0.01) |
| Zhou, 2003 | Yes  General  No | 26  37  8 | 76  91  9 | Significance different  (χ^2^ =20.89, p＜0.01) |

A14-attitude of future career

| Author | Group | Depression | non-depression | Results |
| --- | --- | --- | --- | --- |
| Zhai, 2006 | Optimistic  Relatively optimistic  Relatively pessimistic  pessimistic | 7  34  56  17 | 36  208  133  18 | Significance different  (χ^2^ =30.29, p＜0.01) |

A15-academic pressure

| Author | Group | Depression | non-depression | Results |
| --- | --- | --- | --- | --- |
| Zhou, 2003 | Big  Normal  small | 30  40  1 | 57  113  6 | Significance different  (χ^2^ =6.40, p＜0.05) |

A16-clinical internship

| Author | Group | Depression | non-depression | Results |
| --- | --- | --- | --- | --- |
| Pan, 2016 | Yes  No  Missing | 221  1474 | 978  6090 | Significance different  (χ^2^ value is not stated, p=0.339) |

A17-retaking college entrance examination in senior

| Author | Group | | Depression | non-depression | Results |
| --- | --- | --- | --- | --- | --- |
| Pan, 2016 | | Yes  No | 510  1196 | 1673  5471 | No significance different  (χ^2^ value is not stated, p＜0.001)  Yes (95%CI:0.96-1.27, OR: 1.10) |

A18-personality factor score

| Author | Group | Total score | Results |
| --- | --- | --- | --- |
| Lin, 2018 | Eysenck E factor  Eysenck N factor  Eysenck P factor  Eysenck L factor | 49.9984±10.0142  48.9437±10.0905  50.1919±10.8017  50.1070±10.0051 | Significance different  L is reference group  E (t=-2.727, p=0.007)  N (t=6.598, p＜0.001)  P (t=2.557, p=0.011) |

A19-adaptability factor score

| Author | Group | Total score | Results |
| --- | --- | --- | --- |
| Lin, 2018 | Continuous data | 203.2990±27.36697 | Significance different  (T=-3.706, p＜0.001) |

B1-father’s education

| Author | Group | Depression | Non-depression | Results |
| --- | --- | --- | --- | --- |
| Pan, 2016 | Primary school or illiterate  Junior middle school or equivalent  Senior middle school or equivalent  College or postgraduate  Missing | 393  590  432  326 | 1034  2344  2114  1749  28 | Significance different  (χ^2^ value is not stated, p＜0.001)  PSI (95%CI: 1.13-1.80, OR=1.43, adjusted)  JMSE (95%CI: 0.93-1.40, OR=1.14, adjusted)  SMSE (95%CI: 0.79-1.16, OR=0.95, adjusted) |

B5-monthly household income per capita (Yuan)

| Author | Group | depression | Non-depression | Results |
| --- | --- | --- | --- | --- |
| Pan, 2016 | ＜800  800-1999  2000-2999  ＞2999  Missing | 509  586  301  336 | 1454  2353  1500  1881  90 | Significance different  (χ^2^ value is not stated, p＜0.001)  ＜800 (95%CI: 1.37-2.04, OR=1.67, adjusted)  800-1999 (95%CI: 1.08-1.55, OR=1.29, adjusted)  2000-2999(95%CI: 0.90-1.33, OR=1.09, adjusted) |
| Sun, 2011 | Good  Moderate  Poor | 20  859  550 | 167  5771  2776 | Significance different  (χ^2^ =59.34, p＜0.01)  Good (95%CI: 0.45-1,14, OR=0.36)  Moderate (95%CI: 0.57-0.71, OR=0.64) |
| Zhou, 2003 | ＜500  ＜1000  ＜1500  ＜2000  ＞2000 | 11  17  15  9  19 | 27  44  35  25  45 | Significance different  (χ^2^ =0.228, p＞0.05) |

B6-family characteristics

| Author | Group | depression | Non-depression | Results |
| --- | --- | --- | --- | --- |
| Sun, 2011 | Intact family  Non-intact family | 1334  92 | 8202  512 | No significance different  (χ^2^ =5.12, p＞0.05) |

B7-life events score

| Author | Group | Total score | Results |
| --- | --- | --- | --- |
| Lin, 2018 | Continuous data | 55.59±21.426 | Significance different  (T=3.683, p≤0.001) |

C1-social support score

| Author | Group | Total score | Results |
| --- | --- | --- | --- |
| Lin, 2018 | Continuous data | 67.21±12.319 | Significance different  (T=-4.3145, p≤0.001) |

**Anxiety and non-anxiety**

A1-gender

| Author | Group | anxiety | Non-anxiety | Results |
| --- | --- | --- | --- | --- |
| Li, 2015 | Male  Female | 92  80 | 252  424 | Significance different  (χ^2^ =7.472, p=0.006) |
| Li, 2015 | Male  Female | 202  188 | 402  702 | Significance different  (χ^2^ =28.312, p＜0.01)  Female (95%CI: 0.508-0.850, OR=0.658) |
| Qu, 2014 | Male  Female | 47  169 | 83  238 | No significance different  (χ^2^ =1.22, p=0.27) |
| Ruan, 2011 | Male  Female | 51  77 | 146  236 | No significance different  (χ^2^ is not stated, p=0.744) |
| Sun ,2011 | Male  Female | 560  866 | 4120  4594 | Significance different  (χ^2^ =33.48, p＜0.01)  Female (95%CI: 1.33-1.69, OR=1.50) |
| Feng, 2010 | Male  Female | 140  136 | 294  397 | Significance different  (χ^2^ =8.51, p＜0.05) |
| Liu, 2009 | Male  Female | 53  106 | 22  14 | Significance different  (χ^2^ =10.0057, p=0.007) |
| Liang, 2007 | Male  Female | 105  209 | 103  198 | No significance different  (χ^2^ =5.19, p=0.26) |
| Yang, 2006 | Male  Female | 23  18 | 231  208 | No significance different  (χ^2^ =0.l821, p=0.6696) |

A2-grade

| Author | Group | anxiety | Non-anxiety | Results |
| --- | --- | --- | --- | --- |
| Li, 2015 | Sophomore  Junior | 80  64 | 286  418 | Significance different  (χ^2^ =5.432, p=0.019) |
| Li, 2015 | Freshman  Sophomore(the second grade)  Sophomore(the third grade)  Junior  Senior | 54  55  72  80  129 | 242  235  231  224  172 | Significance different  (χ^2^ =61.806, p＜0.01) |
| Ruan, 2011 | Freshmen  Middle  Senior | 19  70  39 | 53  224  105 | No significance different  (χ^2^value is not stated, p=0.717) |
| Sun ,2011 | Freshman  Sophomore | 637  789 | 4878  3836 | Significance different  (χ^2^ =68.76, p＜0.01)  Freshmen (95%CI: 0.69-0.89, OR=0.79) |
| Feng, 2010 | Freshman  Sophomore(the second grade)  Sophomore(the third grade)  Junior  Senior | 63  51  53  53  96 | 142  198  199  184  181 | Significance different  (χ^2^=11,51, p＜0.05) |
| Liu, 2009 | Junior  Senior | 12  24 | 86  73 | Significance different  (χ^2^=8.660, p=0.013) |
| Zhai, 2006 | Freshmen  Sophomore  Junior  senior | 5  26  11  4 | 171  138  113  41 | Significance different  (χ^2^=17.50, p＜0.01) |

A3-age

| Author | Group | anxiety | Non-anxiety | Results |
| --- | --- | --- | --- | --- |
| Sun, 2011 | 15-19  20-25 | 570  789 | 4070  4644 | Significance different  (χ^2^ =22.85, p＜0.01) |

A43-ethnicity

| Author | Group | anxiety | Non-anxiety | Results |
| --- | --- | --- | --- | --- |
| Feng, 2010 | Han nationality  Minority nationality | 77  199 | 135  556 | Significance different  (χ^2^ =8.69, p＜0.05) |
| Liang, 2007 | Han nationality  Zhuang nationality  Miao nationality  Yao nationality  Dong nationality  others | 195  97  1  6  1  1 | 180  114  2  11  3  4 | Significance different  (χ^2^ =12.37, p=0.90) |

A5-house registry

| Author | Group | anxiety | Non-anxiety | Results |
| --- | --- | --- | --- | --- |
| Li, 2015 | Urban  Rural | 56  114 | 312  366 | Significance different  (χ^2^ =4.721, p=0.030) |
| Li, 2015 | Urban  Rural | 92  264 | 298  840 | No significance different  (χ^2^ =0.017, p＞0.05) |
| Ruan, 2011 | Urban  Rural | 65  63 | 231  141 | Significance different  (χ^2^ value is not stated, p=0.025) |
| Feng, 2010 | Urban  Rural | 199  77 | 429  262 | Significance different  (χ^2^ =9.44, p＜0.05) |
| Liang, 2007 | Urban  Rural | 70  243 | 65  236 | No significance different  (χ^2^ =2.34, p=0.67) |

A6-current major

| Author | Group | anxiety | Non-anxiety | Results |
| --- | --- | --- | --- | --- |
| Liu, 2009 | Clinical medicine  Medical engineering  Nursing  Cognitive Impairment | 42  42  33  42 | 6  7  17  6 | Significance different  (χ^2^ =10.106, p=0.018) |
| Liang, 2007 | Clinical major  Nursing major  Preventive medicine major  Oral medicine | 154  77  55  9 | 174  70  55  21 | No significance different  (χ^2^ =15.72, p=0.20) |

A7-school

| Author | Group | anxiety | Non-anxiety | Results |
| --- | --- | --- | --- | --- |
| Sun, 2011 | College 1  College 2  College 3 | 315  724  387 | 2589  3537  2588 | Significance different  (χ^2^ =60.70, p＜0.01)  College 1 (95%CI: 0.50-0.67, OR=0.58)  College 2 (95%CI: 0.59-0.78, OR=0.68) |

A11-no sibling

| Author | Group | anxiety | Non-anxiety | Results |
| --- | --- | --- | --- | --- |
| Li, 2015 | Yes  No | 36  96 | 126  550 | Significance different  (χ^2^ =4.721, p=0.030) |
| Li, 2015 | Yes  No | 67  323 | 178  926 | No significance different  (χ^2^ =0.235, p＞0.05) |
| Liang, 2007 | Yes  No | 22  275 | 38  280 | No significance different  (χ^2^ =5.81, p=0.21) |

A13-satisfaction with current major

| Author | Group | anxiety | Non-anxiety | Results |
| --- | --- | --- | --- | --- |
| Li, 2015 | Very satisfied  Relatively satisfied  In general  Relatively unsatisfied  Very unsatisfied | 38  50  38  6  10 | 110  342  192  18  14 | No significance different  (χ^2^ =6.125, p=0.19) |
| Sun, 2011 | Very satisfied  Satisfied  Non-satisfied | 411  854  161 | 3302  4884  528 | Significance different  (χ^2^ =86.34, p＜0.01)  Very satisfied (95%CI: 0.50-0.76, OR=0.62)  Satisfied (95%CI: 0.58-0.85, OR=0.70) |
| Zhai, 2006 | Very satisfied  Relatively satisfied  Relatively unsatisfied  Very unsatisfied | 14  13  13  8 | 194  168  98  21 | Significance different  (χ^2^ =15.13, p＜0.05) |

A14-the attitude of future career

| Author | Group | anxiety | Non-anxiety | Results |
| --- | --- | --- | --- | --- |
| Zhai, 2006 | Optimistic  Relatively optimistic  Relatively pessimistic  Pessimistic | 4  14  18  10 | 39  228  171  25 | Significance different  (χ^2^ =19.40, p＜0.01) |

A15-the frequency of academic pressure

| Author | Group | anxiety | Non-anxiety | Results |
| --- | --- | --- | --- | --- |
| Li, 2015 | Never  Occasionally  Often  Always | 4  62  40  54 | 36  394  166  52 | Significance different  (χ^2^ =6.125, p=0.047) |

A20-school Loan

| Author | Group | anxiety | Non-anxiety | Results |
| --- | --- | --- | --- | --- |
| Liang, 2007 | Yes  No | 41  260 | 30  279 | No significance different  (χ^2^ =9.89, p=0.78) |

A21-the ability to deal with interpersonal relationship

| Author | Group | anxiety | Non-anxiety | Results |
| --- | --- | --- | --- | --- |
| Li, 2015 | Very good  Relatively good  Relatively bad  Very bad | 2  92  74  4 | 30  460  198  8 | No significance different  (χ^2^ =11.26, p=0.01) |

B1-father’s education level

| Author | Group | anxiety | Non-anxiety | Results |
| --- | --- | --- | --- | --- |
| Li, 2015 | Primary school or illiterate  Middle school or technical secondary school  Above the junior college | 28  118  26 | 68  452  156 | No Significance different  (χ^2^ =4.398, p=0.111) |
| Ruan, 2011 | Below the senior school  Above the senior school | 80  48 | 246  136 | No Significance different  (χ^2^ value is not stated , p=0.699) |

B2-mother’s education level

| Author | Group | anxiety | Non-anxiety | Results |
| --- | --- | --- | --- | --- |
| Li, 2015 | Primary school or illiterate  Middle school or technical secondary school  Above the junior college | 64  92  14 | 158  412  106 | Significance different  (χ^2^ =8.46, p=0.015) |
| Ruan, 2011 | Below the senior school  Above the senior school | 54  74 | 163  219 | No significance different  (χ^2^ value is not stated, p=0.106) |

B3-father’s employment

| Author | Group | anxiety | Non-anxiety | Results |
| --- | --- | --- | --- | --- |
| Ruan, 2011 | Yes  No | 32  96 | 89  293 | No significance different  (χ^2^value is not stated, p=0.695) |

B4-mother’s employment

| Author | Group | anxiety | Non-anxiety | Results |
| --- | --- | --- | --- | --- |
| Ruan, 2011 | Yes  No | 20  108 | 51  331 | No significance different  (χ^2^value is not stated, p=0.520) |

B5-financial status

| Author | Group | anxiety | Non-anxiety | Results |
| --- | --- | --- | --- | --- |
| Li, 2015 | High  Middle  Low  Minima | 2  94  54  22 | 20  444  172  40 | Significance different  (χ^2^ =7.508, p=0.044) |
| Sun, 2011 | Good  Moderate  Poor | 20  856  550 | 167 5771  2776 | Significance different  (χ^2^ =59.34, p＜0.01)  Good (95%CI: 0.47-1.20, OR=0.75)  Moderate (95%CI: 0.65-0.83, OR=0.70) |

B6-family characteristics

| Author | Group | anxiety | Non-anxiety | Results |
| --- | --- | --- | --- | --- |
| Sun, 2011 | Intact family  Non-traditional family | 1334  92 | 8202  512 | No significance different  (χ^2^=5.12, p＞0.05) |

**Appendix 5 The Significant Results**

The summary of the factors influencing the depression and anxiety of medical students is listed in table 1 and table 2

Table 1 Summary of articles reporting factors influencing depression

| Author/year | Mental problem | Key finding (significant results) | Factors |
| --- | --- | --- | --- |
| Lin, 2018 | Depression | The percentage of depression in medical students is 13.1%;  Factors:  1. The student’s adaptability score, social support score and the E personality students negatively affect depression.  2. Negative events score, N personality and P personality students are positively affect depression.  3. The sophomore (the second grade students) students, the senior students and male students are more depressive than their peers. | IF  SEF  EF |
| Li, 2017 | Depression | The percentage of depression in medical students is 36.11%;  Factors:  The minority nationality, non-single child, rural students, the senior students and western college medical students are more depressive than their peers. | IF |
| Pan, 2016 | Depression | The percentage of depression in medical students is 19.9%;  Factors:  1. Male, low monthly income per capita, father’s poor education background, and higher year of study medical students are more depressive than their peers.  2. Medical students in comprehensive universities are more likely to be depressive compared with those from medical universities.  3. The smoker, alcoholic, insomniacs and hospitalization or medication for one week or more in the last four weeks experience a higher depressive symptoms among medical students | IF  SEF |
| Zhai, 2014 | Depression& Anxiety | The percentage of depression in medical students is 30.4%;  1. The pharmacy major students are more depressive than their peers.  2. The bad grade students are more depressive than their peers.  3. The bad relationship students are more depressive their peers. | IF  SEF |
| Guo, 2014 | Depression | The percentage of depression in medical students is 52.0%;  1. Nursing students are more depressive than their peers.  2. Bad self –evaluation students, few good friends, low association participate rate, low interest in current major, bad employment prospection, high academic pressure, family pressure and family conflict are associated with high depressive symptoms among medical students. | IF  SEF |
| Ren, 2013 | Depression | The percentage of depression in medical students is 24.4%;  Family history of depression, smoking and drinking habits, introverted personality and interpersonal maladjustment are risk factors for depression among medical students. | IF |
| Sun, 2011 | Depression& Anxiety | The percentage of depression in medical students is 16.8%;  1. The second-year students had higher levels of psychological  symptoms than the first-year students  2. Social support, family function, and all dimensions were significantly negatively associated with depression and anxiety symptoms.  3. Less social support, poor family function, the second year students, and unsatisfactory specialty were associated with more psychological symptoms. | IF  SEF  EF |
| Shang, 2009 | Depression | The percentage of depression in medical students is 39.3%;  Different major, bad family financial status, bad classmate relationship, bad psychological quality, suicide ideation, suicide plan, suicide action are related to higher depression. | IF  SEF |
| Li, 2009 | Depression | The percentage of depression in medical students is 76.21%;  Male, low monthly income per capita, father’s poor education background, and higher year of study medical students are more depressive than their peers. | IF  SEF |
| He, 2009 | Depression& Anxiety | The percentage of depression in medical students is 25.32%;  Senior students and non-single students are more depressive than their peers. | IF |
| Qu,2006 | Depression& Anxiety | The percentage of depression in medical students is 22.40%;  1. Sophomore students are more depressive than their peers.  2. Rural students are more depressive than their peers.  3. Bad future employment anticipation and low current major satisfaction rate students are more depressive than their peers. | IF |
| Yang, 2005 | Depression& Anxiety | The percentage of depression in medical students is 16.46%;  Bad sleeping condition is related to high depressive symptoms. | IF |
| Zhou, 2003 | Depression | The percentage of depression in medical students is 40.34%;  1. Male students are more depressive than their peers.  2. High academic pressure students are more depressive than their peers.  3. Low interest of current major students are more depressive than their peers. | IF |
| Yu, 2001 | Depression | The percentage of depression in medical students is 30.50%  1. Male students are more depressive than their peers  2. Students who feel loneness is tried to study, experience bad sleeping condition are more depressive than their peers.  3. Academic pressure students are more depressive than their peers. | IF |
| Wu, 2000 | Depression | The percentage of depression in medical students is 43.00%  Being discrimination, being understood are connected with higher level of depression. | IF  SEF |

Table 2 Summary of articles reporting factors influencing anxiety

| Author/year | Mental problem | Key finding (significant results) | Factors |
| --- | --- | --- | --- |
| Li,2015 | Anxiety | The percentage of anxiety in medical students is 20.3%;  Factors:  1. The male students, sophomore students, and rural students are more anxious than their peers.  2. Low mother education background, bad financial status students are more anxious than their peers.  3. High academic pressure and bad interpersonal relationship dealer are more anxious than their peers. | IF  SEF |
| Li, 2015 | Anxiety | The percentage of anxiety in medical students is 26.1%;  1. The male students, higher grade students are more anxious than their peers  2. Stress score is positively affect anxiety. | IF |
| Zhai, 2014 | Depression& Anxiety | The percentage of anxiety in medical students is 40.4%;  The bad family financial status students is associated with higher anxious symptoms. | IF  SEF |
| Ruan, 2011 | Anxiety | The percentage of anxiety in medical students is 25.1%;  1. The rural students are more anxious than their urban peers.  2. The higher parental education, the lower the children anxiety.  3. Self-evaluation, professional preferences, adaptability, health threats, two-way choice, social pressure only child and bad mental quality is related to higher anxiety. | IF  SEF |
| Sun, 2011 | Depression& Anxiety | The percentage of anxiety in medical students is 14.1%;  1. Female students were more likely to have anxiety.  2. The second-year students had higher levels of psychological symptoms than the first-year students.  3. Social support, family function, and all dimensions were significantly negatively associated with anxiety and anxiety symptoms.  4. Less social support, poor family function, the second year students, and unsatisfactory specialty were associated with frequent psychological symptoms. | IF  SEF  EF |
| Feng, 2010 | Anxiety | The percentage of anxiety in medical students is 28.54%;  Male students, han nationality students, senior students, and rural students are more anxious than their peers. | IF |
| He, 2009 | Depression& Anxiety | The percentage of anxiety in medical students is 25.32%;  Senior students and non-single students are more anxious than their peers. | IF |
| Liu, 2009 | Anxiety | The percentage of anxiety in medical students is 88.30%;  Male students, senior students, clinical discipline of Chinese and western integrative medicine major students are more anxious than their peers. | IF |
| Liang, 2007 | Anxiety | The percentage of anxiety in medical students is 16.60%;  Non-single child students are more anxious than their peers. | IF |
| Qu,2006 | Depression& Anxiety | The percentage of anxiety in medical students is 9.03%;  1. Sophomore students are more anxious than their peers.  2. Bad future employment anticipation and low current major satisfaction rate students are more anxious than their peers. | IF |
| Yang, 2005 | Depression& Anxiety | The percentage of anxiety in medical students is 8.54%;  Bad sleeping condition is related to high anxious symptoms. | IF |

**Appendix 6 EDITORIAL CERTIFICATET**


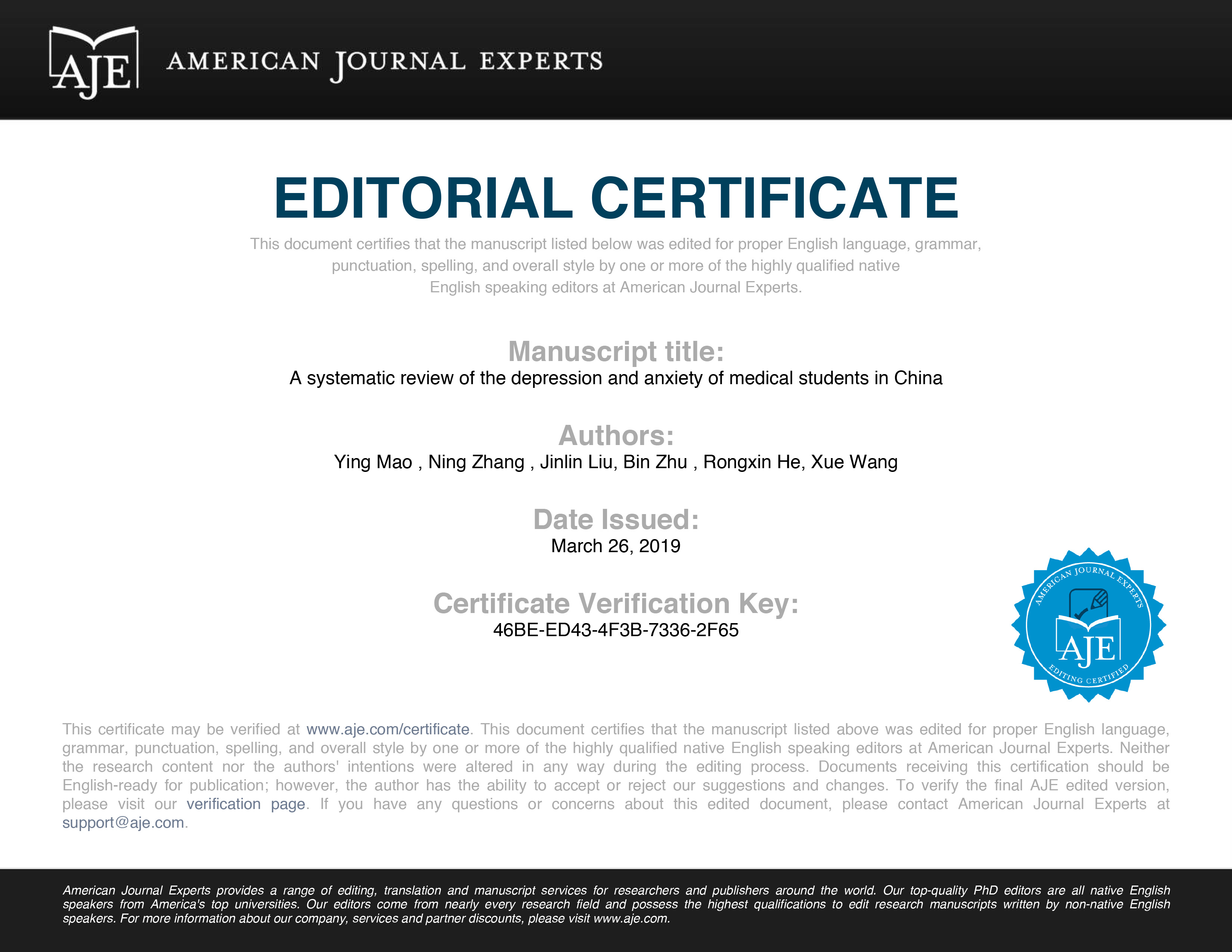

Supplement: Supplementary file 1 — The Search Strategy & The extracted determinants & The distribution of determinants & The Extracted Determinants & The Significant Results. (DOCX 944 kb) [file 12909_2019_1744_MOESM1_ESM.docx]
